# Dietary *Enterococcus faecium* NCIMB 10415 and Zinc Oxide Stimulate Immune Reactions to Trivalent Influenza Vaccination in Pigs but Do Not Affect Virological Response upon Challenge Infection

**DOI:** 10.1371/journal.pone.0087007

**Published:** 2014-01-28

**Authors:** Zhenya Wang, Michael Burwinkel, Weidong Chai, Elke Lange, Ulrike Blohm, Angele Breithaupt, Bernd Hoffmann, Sven Twardziok, Juliane Rieger, Pawel Janczyk, Robert Pieper, Nikolaus Osterrieder

**Affiliations:** 1 Institut für Virologie, Freie Universität Berlin, Berlin, Germany; 2 Abteilung für experimentelle Tierhaltung und Biosicherheit, Friedrich-Loeffler-Institut, Greifswald-Insel Riems, Germany; 3 Institut für Immunologie, Friedrich-Loeffler-Institut, Greifswald-Insel Riems, Germany; 4 Institut für Veterinärpathologie, Freie Universität Berlin, Berlin, Germany; 5 Institut für Virusdiagnostik, Friedrich-Loeffler-Institut, Greifswald-Insel Riems, Germany; 6 Molekularbiologie und Bioinformatik, Charité Universitätsmedizin Berlin, Berlin, Germany; 7 Institut für Veterinäranatomie, Freie Universität Berlin, Berlin, Germany; 8 Bundesinstitut für Risikobewertung, Abteilung für Biologische Sicherheit, Fachgruppe für Molekulare Diagnostik und Genetik, Berlin, Germany; 9 Institut für Tierernährung, Freie Universität Berlin, Berlin, Germany; University of Minnesota, United States of America

## Abstract

Swine influenza viruses (SIV) regularly cause significant disease in pigs worldwide. Since there is no causative treatment of SIV, we tested if probiotic *Enterococcus (E.) faecium* NCIMB 10415 or zinc (Zn) oxide as feed supplements provide beneficial effects upon SIV infection in piglets. Seventy-two weaned piglets were fed three different diets containing either *E. faecium* or different levels of Zn (2500 ppm, Zn^high^; 50 ppm, Zn^low^). Half of the piglets were vaccinated intramuscularly (VAC) twice with an inactivated trivalent SIV vaccine, while all piglets were then infected intranasally with H3N2 SIV. Significantly higher weekly weight gains were observed in the *E. faecium* group before virus infection, and piglets in Zn^high^ and *E. faecium* groups gained weight after infection while those in the control group (Zn^low^) lost weight. Using ELISA, we found significantly higher H3N2-specific antibody levels in the *E. faecium*+VAC group 2 days before and at the day of challenge infection as well as at 4 and 6 days after challenge infection. Higher hemagglutination inhibition (HI) titers were also observed in the Zn^high^+VAC and *E. faecium*+VAC groups at 0, 1 and 4 days after infection. However, there were no significant differences in virus shedding and lung lesions between the dietary groups. Using flow cytometry analysis significantly higher activated T helper cells and cytotoxic T lymphocyte percentages in the PBMCs were detected in the Zn^high^ and *E. faecium* groups at single time points after infection compared to the Zn^low^ control group, but no prolonged effect was found. In the BAL cells no influence of dietary supplementation on immune cell percentages could be detected. Our results suggest that feeding high doses of zinc oxide and particularly *E. faecium* could beneficially influence humoral immune responses after vaccination and recovery from SIV infection, but not affect virus shedding and lung pathology.

## Introduction

Swine influenza virus (SIV) is a major cause of acute respiratory infections of pig populations worldwide. The causative agents are type A influenza viruses, mainly of the H1N1, H3N2, or H1N2 subtypes. The main route of transmission is through direct contact between infected and uninfected animals, close contacts being particularly common during animal transport. Intensive farming may also increase the risk of transmission as pigs are raised in production units with high animal densities [1], [2]. SIV infections result in fever, sneezing, coughing, difficulty in breathing, decreased appetite resulting in weight loss and poor growth [1]. SIV can cause significant production losses, especially when complicated by secondary infections.

Porcine respiratory tract epithelial cells express sialic acid receptors utilized by both avian and mammalian influenza viruses. Pigs are, therefore, considered “mixing vessels” for new human-avian influenza A virus reassortants with the potential to cause significant respiratory disease or even pandemics in humans [3]. Thus, the control of SIV is of economic importance but also paramount for public health. Since there currently is no licensed antiviral drug available for pigs, and no sterile immunity is achieved with current vaccines, a positive effect on prevention and/or course of clinical disease achieved through nutritional supplementation would be highly useful.

The effect of zinc (Zn) and various probiotic bacteria on the course of bacterial infections in pigs have been studied intensively [4]–[6]. However, published information on the effect of feed supplements with respect to virus infections is scarce [7], [8]. Probiotic bacteria, as a part of gut microbiome, are reported to promote host defenses and to modulate immune functions [9]. There is evidence that some specific probiotics can alter monocyte and natural killer cell function. Evidence is also accumulating that some probiotics can boost antibody responses to orally and systemically administered vaccines [10], [11]. *E. faecium* NCIMB 10415 is authorized in the EU as a probiotic feed additive for pigs and seems a suitable probiotic that allows us to study possible antiviral effects. It has been demonstrated that this *E. faecium* strain modulates the intestinal immune system in sows and piglets and that it affects shedding of porcine enteric viruses [12], [13]. *In vitro* experiments also showed direct antiviral effects of *E. faecium* against enteric and non-enteric viruses. The potential mechanisms include pathogen exclusion by means of competition for attachment as well the induction of cytokines and signaling molecules which might stimulate host-cell immune defense [14], [15].

Zn is an essential trace element and a cofactor of more than 300 enzymes of all classes. To cover the pig’s requirement of about 50 ppm [16], it is provided as dietary supplement and added to the diet mostly as Zn oxide (ZnO). In addition, it has also been shown that feeding high ZnO levels (2000 to 3000 ppm) to piglets stimulated growth and prevented post-weaning diarrhea [17], [18]. However, for grower/finisher pigs high levels of zinc are typically not sustained, as zinc toxicity is related to dietary level and duration of feeding [16]. Published information on antiviral Zn effects against virus is available from cell culture work and nutritional studies in humans, but less so from studies involving livestock [19], [20]. In humans, Zn was utilized frequently in attempts to treat various virus infections or aid in their prophylaxis. Some results suggest that Zn can directly interact with viral structural components and influence virus replication. It is also widely accepted that Zn affects immune responses on the cellular level as well as on the level of the recipient organism [21]. In cell culture studies, high Zn concentrations and the addition of compounds that stimulate cellular import of Zn were found to inhibit the replication of various RNA viruses, including influenza virus [22].

Since data on the efficacy of probiotic treatment or Zn supplementation on virus infection *in vivo* is limited, especially with respect to extraintestinal effects of the feed supplements, the aim of this study was to explore systemic effects of *E. faecium* and high level Zn oxide feeding on SIV vaccination and infection. We report on clearly demonstrable systemic effects of such treatments, especially with regard to clinical parameters and humoral immune responses that are increased following *E. faecium* and Zn^high^ supplementation.

## Materials and Methods

### Virus, Vaccine, Feed Additives

Influenza A virus (A/swine/Bissendorf/IDT1864/03 (H3N2)) was used for challenge infections. Viral stocks were produced in Madin-Darby canine kidney (MDCK) cells. The inactivated, trivalent vaccine Respiporc® Flu3 (IDT Biologika GmbH, Dessau-Rosslau, Germany), which contains the three main swine influenza subtypes H1N1 (Haselünne/IDT2617/2003) (H3N2), H1N2 (Bakum/1832/2000) and H3N2 (Bakum/IDT1769/2003), was used in our study. Probiotic *E. faecium* NCIMB 10415 was applied as a commercial probiotic feed additive (Cylactin® LBC ME10, DSM nutritional products Ltd, Kaiseraugst, Switzerland) in a microencapsulated form and mixed to the diets of weaned piglets at a concentration of 1×10^9^ colony forming units (CFU)/kg feed. ZnO was either added at a high dose (Zn^high^: 2500 mg/kg diets (pharmacological level) until the age of 56 days and then switched to a medium dose (Zn^med^: 150 mg/kg diet (max. allowed EU level), or no additional ZnO was added (Zn^low^: 50 mg/kg diet). The Zn^low^ diet represents the regular feed of the animals and the Zn^low^ group, therefore, was considered the control throughout the paper.

### Animals and Experimental Setup

All animal experimentation was approved by the local animal welfare authority (Landesamt für Landwirtschaft, Lebensmittelsicherheit und Fischerei, Rostock, Mecklenburg-Vorpommern, Germany) under the registration number 44/12. Piglets (n = 72) were raised at the Institut für Tierernährung, Freie Universität Berlin and weaned at the age of 28 days of age. Pigs were then randomly assigned to three different diets (Zn^low^, Zn^high^ or *E. faecium*) and kept in groups of 6 (2 pens per diet). High Zn levels were fed only until the age of 56 days in order to avoid toxic effects and the diet was then reduced to medium levels (Zn^med^). Half of the piglets (one pen per treatment) were vaccinated intramuscularly (VAC) twice on day 35 and 56 with the commercial SIV vaccine. In total, there were 6 treatment groups containing 12 piglets each. Five days before virus infection, all piglets were transported to the BSL3* facility at the Friedrich-Loeffler-Institut, Insel Riems, where they were housed in HEPA-filtered isolation units at a constant 27°C. All pigs were tested for the presence of SIV antibodies by a commercially available ELISA targeting the viral nucleoprotein (ID Screen® Influenza A competition, ID.vet, Grabels, France) prior to infection. At 63 days of age, all piglets were inoculated by the intranasal route with 2×1 ml of SIV H3N2 with a titer of 10^6.3^ TCID_50_/ml using a LMA MAD™ intranasal mucosal atomization device (Teleflex Medical GmbH, Kernen, Germany). Half of the piglets from each group were killed on 1 and 6 dpi, respectively, by i.v. injection of 0.1 ml/kg body mass T61® (Intervet Deutschland GmbH, Unterschleißheim, Germany) after intramuscular induction of anesthesia with 20–30 mg/kg body mass ketamine (Ursotamin®, Serumwerk Bernburg AG, Bernburg, Germany and 1–2 mg/kg body mass azaperon (Stresnil™, Janssen-Cilag GmbH, Neuss, Germany).

### Clinical Follow-up and Sampling

During the experiment, animals were clinically monitored daily for the development of clinical signs including fever, fatigue, anorexia, dyspnea and cough. Body weights were recorded weekly after weaning before infection and at necropsy on 1 and 6 dpi after exsanguination. Blood samples were taken daily after the second vaccination for serological analyses. Nasal, buccal and fecal swabs were collected daily for the analysis of virus shedding. At the day of necropsy, samples were taken from the nasal turbinates and lungs (apical, middle and accessory lobes). Samples of all organs were prepared for histological analysis.

### Gross Pathology and Histopathology

At necropsy, the lungs were immediately examined macroscopically and photographs taken for further analysis. For histopathology, small sections of organs were fixed in 10% buffered formalin. Fixed tissues were dehydrated, embedded in paraffin and 5 µm sections were cut for histological examination. Lung sections from the portion most consistently affected by gross lesions (well-demarcated purple dark red areas of tissue consolidation) were stained using a hematoxylin/eosin (H&E) standard staining protocol [23] and examined microscopically. Examination of tissue sections from this study was conducted in a blinded fashion by a veterinary pathologist. Lesion severity was scored by the distribution of lesions within the sections examined as follows: 0 - no visible changes; 1 - mild changes, minimally different from the normal; 2 - moderate changes; 3 - severe and diffusely distributed changes.

### Serology

The development of an influenza virus-specific immune response was analyzed by ID Screen® Influenza A competition ELISA. The optical density (OD) of the reaction was measured at 450 nm (OD_450 nm_) with a microplate reader (Tecan, Crailsheim, Germany). Results were reported as a ratio of the OD_450 nm_ of the sample and the negative control (OD_450 nm_ “S”/OD_450 nm_ “N”) included in the kit (positive cut-off: S/N = 0.55). Hemagglutination inhibition (HI) assay was performed using a solution of 0.5% chicken erythrocytes in 0.9% NaCl and 8 hemagglutinating units of A/swine/Bissendorf/IDT1864. Sera were pretreated with receptor destroying enzyme (Cholera filtrate; Sigma-Aldrich, St. Louis, USA) to remove nonspecific inhibitors and adsorbed onto chicken erythrocytes to remove unspecific agglutinating factors. Tests were performed according to standard procedures in twofold dilutions starting at 1∶20.

### Differential Cell Count

To evaluate changes of cellular composition in the peripheral blood after SIV infection, 150 µl of whole blood were analyzed using an automated XT-2000iV hematology analyser (Sysmex Corporation, Hyogo, Japan) and the number of neutrophils, lymphocytes, and monocytes were determined.

### Viral RNA Quantification from Swabs

Nasal, buccal and fecal swabs were taken, placed in vials containing serum-free cell-culture medium, and stored at −80°C until further analysis. Viral RNA was extracted from nasal and buccal swabs taken at 0, 2, 4, 6 dpi and from fecal swabs at 3 dpi using the MagAttract® DNA Mini M48 Kit (Qiagen, Hilden, Germany) on the KingFisher Flex Magnetic Particle Processors (Thermo Fisher Scientific, Waltham, USA). Swabs were eluted in 1 mL serum-free cell-culture media of which 100 µl were used for RNA extraction into 100 µl AVE buffer. Real-time reverse transcriptase RT-qPCR for quantification of SIV copy numbers was performed using a pan-Influenza A-M1.2 assay [24] and an *in vitro*-transcribed RNA standard.

### Flow Cytometry

Peripheral blood mononuclear cells (PBMCs) were subjected to multicolor immunostaining with porcine cell surface markers for flow cytometry analysis using a BD FACSCanto™ (BD Biosciences, Heidelberg, Germany). Each heparinized blood sample (50 µl) was stained with antibody mix 1 (Table S1). Respective isotypes were also included in the assay. After incubation for 15 min in the dark at 4°C, cells were washed with fluorescence-activated cell sorting (FACS) buffer (0.1% BSA, 0.035% sodium bicarbonate and 0.02% sodium azide in HBSS) and centrifuged for 5 min at 700×g. Then, antibody mix 2, mix 3 or mix 4 were added and cells were washed and centrifuged. After the last wash step, contaminating erythrocytes were lysed by osmosis with distilled water and samples analyzed. Lung mononuclear cells were isolated from freshly euthanized piglets after removal of lungs with trachea and bronchus. The left lungs were lavaged with 50 ml of PBS (pH 7.4) using a flexible tube and the collected bronchoalveolar lavage (BAL) samples were centrifuged at 300×g for 10 min at 4°C. The pellet was resuspended in FACS buffer and stained as described above. Results from flow cytometry were analyzed using FlowJo™ software (Treestar, Ashland, USA). Based on γδ-T cell receptor (gdTCR), CD3, CD4, CD8, CD2 and CD21 staining characteristics, each subpopulation was then further grouped as follows: γδ-T cells (gdTCR^+^CD3^+^CD2^+^CD8^+^); T-helper (Th) cells (gdTCR^−^CD3^+^CD4^+^CD8^−^); activated T helper cells (gdTCR^−^CD3^+^CD4^+^CD8^−^CD25^high^); cytotoxic T lymphocytes (CTLs) (gdTCR^−^CD3^+^CD4^−^CD8^+^); Th/memory cells (gdTCR^−^CD3^+^CD4^+^CD8^+^); natural killer (NK) cells (gdTCR^−^CD3^−^CD4^−^CD8^high^); antibody-forming and/or memory B cells (gdTCR^−^CD3^−^CD2^+^CD21^−^).

### Statistical Analysis

Results were analyzed by a mixed model with fixed effects (time, diet, time*diet (ELISA and HI assay data); diet, vaccination, diet*vaccination (lesion score data); time, diet, vaccination, time*diet, time*vaccination, diet*vaccination, time*diet*vaccination (qRT-PCR, blood count, flow cytometry data)) and one random effect (animal). Post-hoc tests (LSD) were applied in case of significant effects. Calculations were performed with SPSS® Version 21 (IBM, Armonk, NY, USA) and GraphPad Prism 5 (GraphPad Software Inc., La Jolla, CA, USA).

## Results

### Clinical Symptoms and Weight Gains

**Figure 1:**
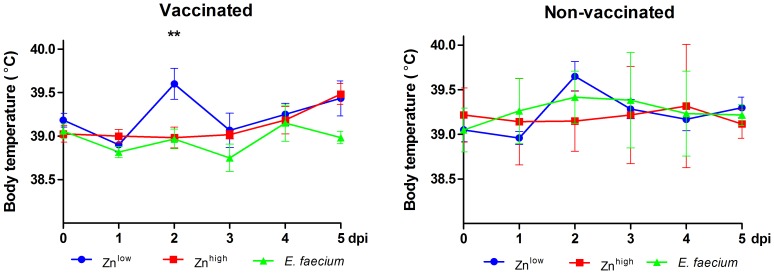
Body temperatures. Individual body temperatures were measured rectally daily after infection. Each bar represents the mean value ± standard deviation from 6 pigs. A significant difference is shown for the *E. faecium*+VAC compared to Zn^low^+VAC group (**: P<0.01).

Clinically, SIV infection caused only mild symptoms (Figure 1) with fever (≥40°C) only sporadically observed. Average body temperatures were lowest in the *E. faecium*+VAC group throughout the observation period after SIV infection, with mean temperatures spiking in the vaccinated Zn^low^ group on 2 dpi (Figure 1). Significantly higher weekly weight gains were recorded before infection in the *E. faecium* groups in the period from 39 to 46 and from 46 to 53 days of age, regardless of vaccination (Figure S1A). Comparing the body weights after exsanguination, mean body weights in all vaccinated and non-vaccinated Zn^high^ and *E. faecium* fed groups increased after SIV infection from 1 dpi to 6 dpi, while the weights declined in the Zn^low^ groups at those time points (Figure S1B).

### Gross and Histopathology

**Figure 2:**
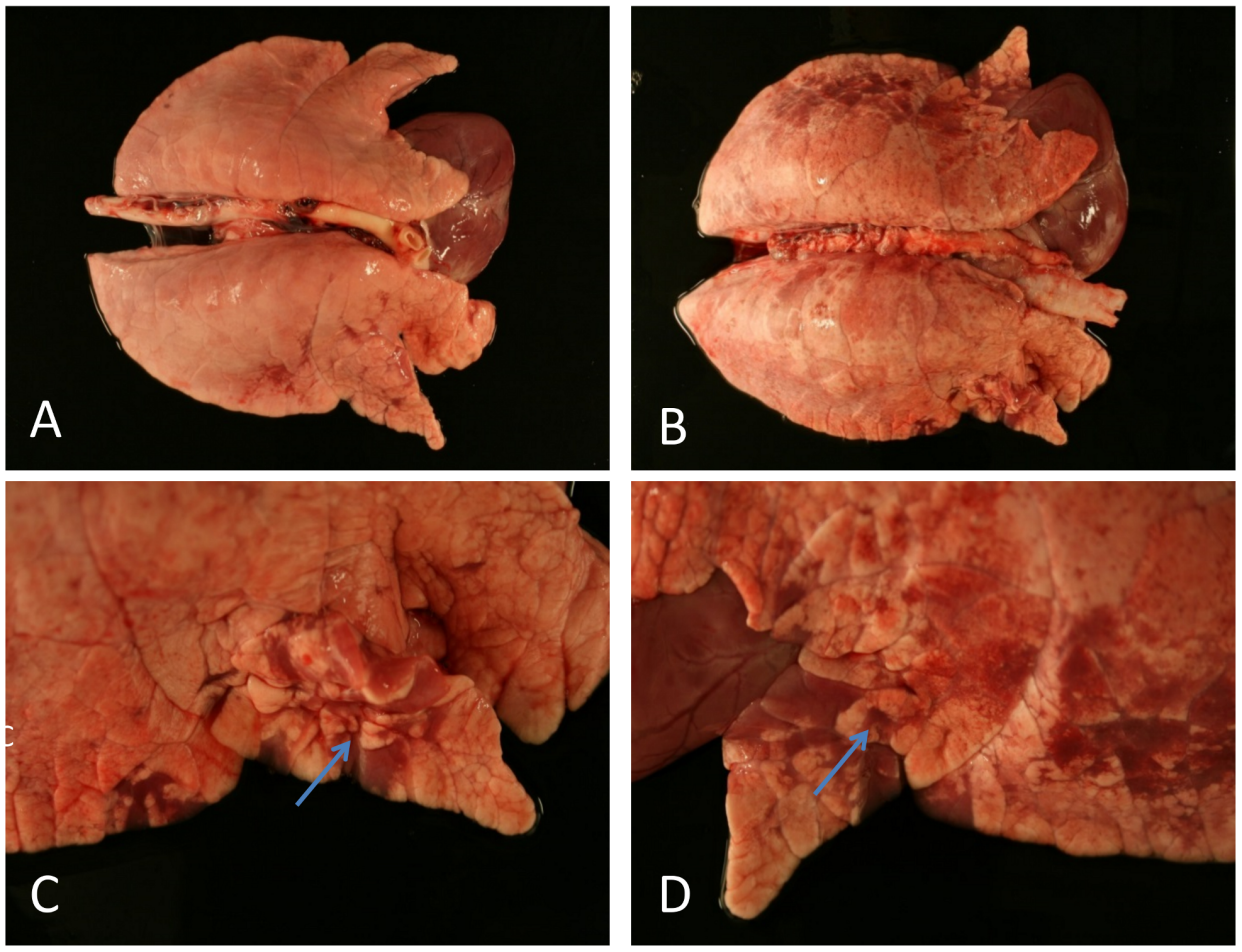
Exemplary gross lesions in lungs after SIV infection. (**A**) Lung from a vaccinated piglet at 6 dpi. (**B**) Lung from a non-vaccinated piglet at 6 dpi. (**C and D**) Detailed pictures of lung B showing focal areas of tissue consolidation (arrows).

**Figure 3:**
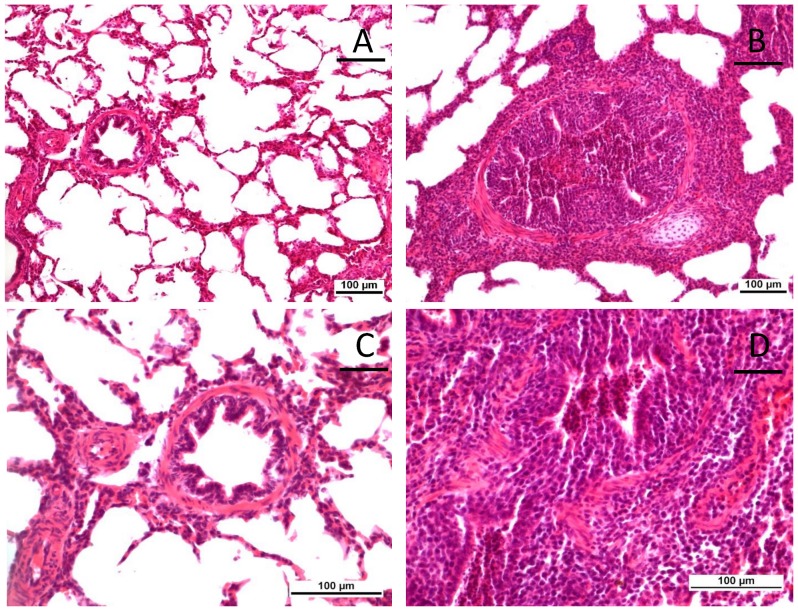
Microscopical examination of lung sections. (**A and C**) H&E stained lung of a vaccinated piglet with normal bronchial epithelial lining and absence of infiltrates of inflammatory cells. (**B and D**) H&E stained lung of a non-vaccinated piglet with extensive infiltration of predominantly lymphocytes in the interstitium and around bronchi and bronchioli.

**Figure 4:**
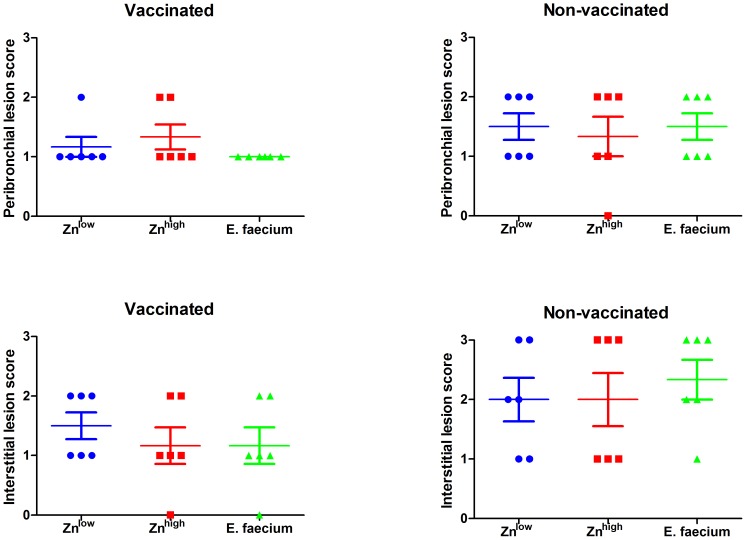
Pathohistological lesion scoring. Scores of lung lesions in the right middle lobes (0 - no visible changes; 1 - mild changes, minimally different from the normal; 2 - moderate changes; 3 - severe and diffusely distributed changes).

Vaccination resulted in reduced frequency of parenchymal consolidation in the lungs of piglets in all diet groups (Figure 2A). Non-vaccinated animals showed more macroscopic (Figure 2B–D) and microscopic lesions at 6 dpi (Figure 3). The right middle lung lobes exhibited the highest frequency and extent of lesions macroscopically; therefore, sections from this lobe were further analyzed and scored after histopathologic examination. Affected pigs presented with mild (score 1) to severe (score 3) interstitial bronchopneumonia that was dominated by lymphocytic infiltration (Figure 4). Sporadically, bronchioles and alveoli contained cellular debris with lymphocytes, fewer histiocytes and scattered neutrophils accompanied by bronchiolar epithelial degeneration and necrosis. In the vaccinated groups, a reduced frequency of moderate (score 2) peribronchial lesions and a prevention of severe (score 3) interstitial lungs lesion was observed. Although no significant differences between different diets were apparent in vaccinated and non-vaccinated animals, vaccinated animals that had received *E. faecium* in the diet showed the lowest lesion scores (Figure 3).

### Virus Shedding

**Figure 5:**
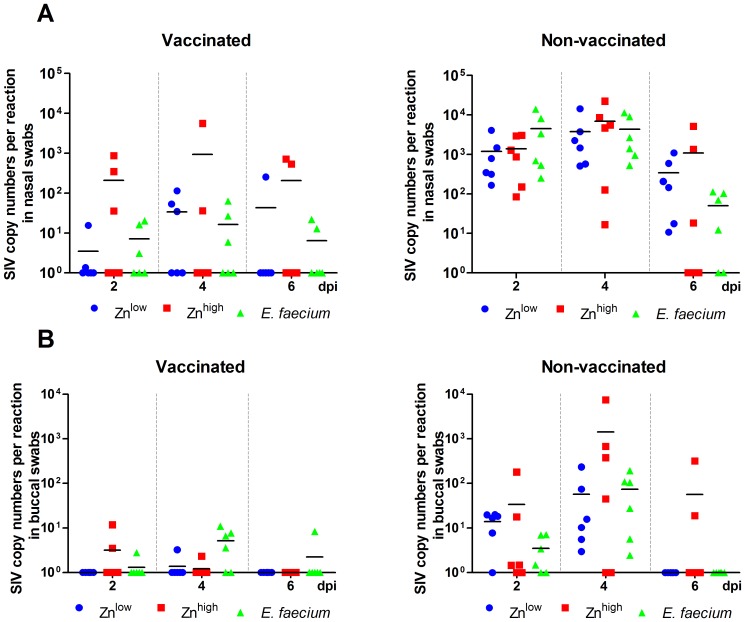
SIV antibody ELISA. SIV-specific antibodies were detected in swine sera by competition ELISA targeting NP from −2 dpi to 6 dpi. The dotted line indicates the threshold above which values are considered positive.

Virus shedding was analyzed by qRT-PCR in nasal and buccal swabs before infection and at 2, 4 and 6 dpi (Figure 5) and in fecal swabs from 3 dpi. No virus genomes were detectable in samples before infection and in the fecal swabs (data not shown). Vaccinated animals had approximately 10- to 100-fold reduced viral loads when compared to non-vaccinated animals in both nasal and buccal swabs (Figure 5). There were no significant differences, however, between dietary treatment groups, although, again, a tendency towards lower virus shedding from the nose was observed for *E. faecium*-treated animals.

### Humoral Immune Responses

**Figure 6:**
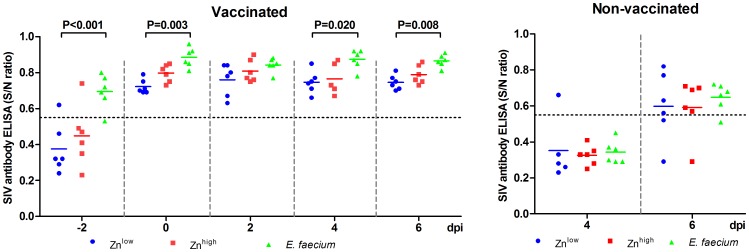
Hemagglutination inhibition (HI) antibody titers. Twofold serum dilutions starting at 1∶20 were examined. Values ≥80 (dotted line) are considered positive.

**Figure 7:**
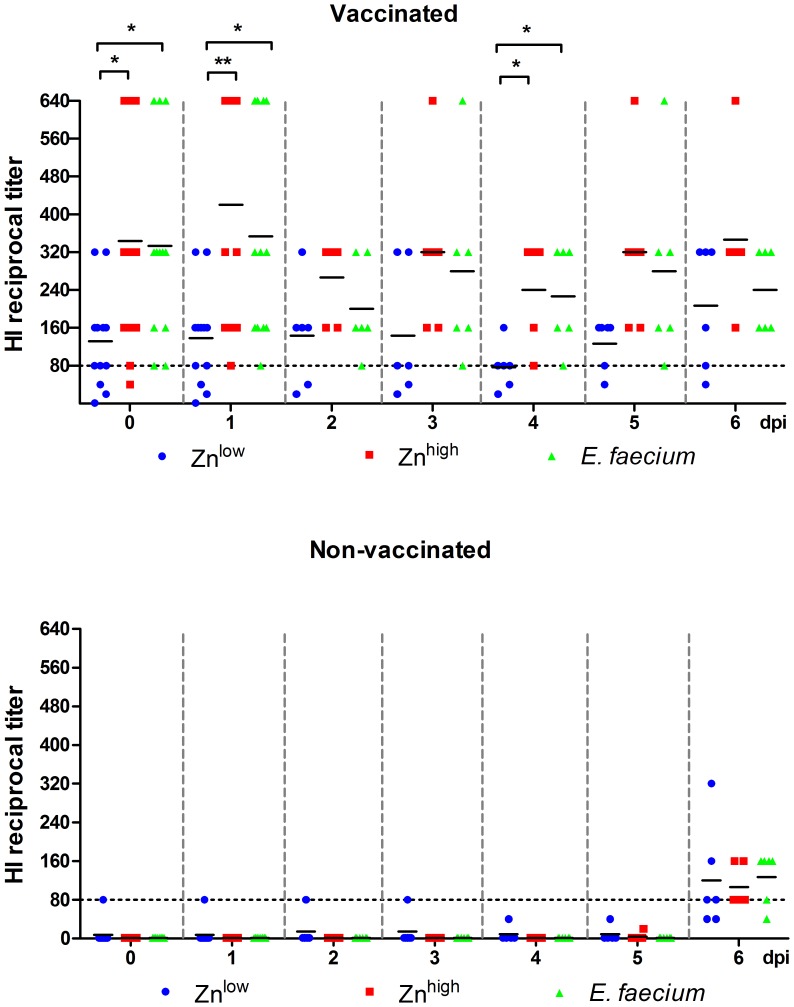
qRT-PCR. Virus shedding in nasal (**A**) and buccal swabs (**B**). SIV genome copy numbers were detected in swab eluates. All swabs taken at the day of infection (0 dpi) were negative (not shown).

All vaccinated piglets had developed antibodies as detected with the NP protein ELISA at the day of infection and 7 days after the second vaccination, (Figure 6). Significantly higher H3N2-specific antibodies could be detected in the *E. faecium*+VAC groups compared to the Zn^low^+VAC groups 2 days before (P<0.001) and on the day of challenge infection (P = 0.008) as well as on 4 (P = 0.038) and 6 dpi (P = 0.017). The ELISA results were confirmed by an HI assay (Figure 7). Again, we found a general effect of feeding *E. faecium* and the Zn^high^ diet in the vaccinated animals. Significantly higher antibody titers were detected in the *E. faecium*+VAC groups on the day of SIV infection (0 dpi, P<0.05), 1 dpi (P<0.05) and 4 dpi (P<0.05). Significantly higher antibodies were also detected in the Zn^high^+VAC groups on the day of SIV infection (P<0.05), 1 dpi (P<0.01) and 4 dpi (P<0.05). For the non-vaccinated piglets, antibodies could barely be detected at 6 dpi by either method.

### Cellular Immune Responses

Hematological parameters in peripheral blood were examined after SIV infection using an automated analyzer. As shown in Table 1, the numbers of monocytes and lymphocytes showed no differences between the groups, whereas reduced numbers of neutrophils were observed in the Zn^high^ groups.

**Table 1:** Blood count.

Cell type	dpi	Zn^low^	Zn^high^	*E. f.*	Zn^low^ +VAC	Zn^high^ +VAC	*E. f.* +VAC
Neutrophils	0	87.6	53.3	83.9	80.2	75.6	76.0
(20–70)		±23.5	±12.2	±33.8	±14.8	±15.5	±11.6
	3	92.8	77.9	94.9	100.3	88.2	109.4
		±12.9	±31.2	±21.9	±28.22	±22.1	±43.9
	6	94.9	84.9	102.3	70.1	86.1	91.6
		±6.9	±40.3	±15.5	±19.1	±19.4	±26.6
Lymphocytes	0	87.8	103.2	97.0	89.3	85.8	94.7
(60–340)		±15.4	±27.2	±35.1	±10.1	±16.8	±9.5
	3	92.0	107.4	94.0	89.1	103.9	111.7
		±13.1	±16.7	±13.3	±23.8	±20.1	±15.6
	6	93.1	86.4	81.1	69.8	89.7	79.7
		±24.3	±21.4	±5.9	±14.0	±12.1	±9.6
Monocytes	0	15.2	14.3	13.4	13.2	14.6	13.1
(0–9)		±7.0	±4.5	±2.5	±3.0	±2.3	±2.6
	3	13.4	17.6	19.1	15.5	17.2	15.2
		±3.7	±4.3	±7.0	±5.3	±3.3	±3.6
	6	17.6	17.1	13.3	12.1	16.8	9.0
		±4.4	±4.2	±2.8	±2.9	±6.0	±2.0

Changes in blood leukocyte distribution (mean numbers (100/µl) ±SD) of different cell types (reference values in brackets) from 6 piglets/group after influenza virus infection.

Flow cytometry of immune cell phenotypes of PBMC subpopulations was performed from 0 dpi to 6 dpi (f2). Virus infection led to a slight decrease in the frequency of Th cells until 6 dpi. In contrast, increased percentages of CTLs, Th/memory cells, antibody-forming and/or memory B cells, and NK cells were observed until 6 dpi in both vaccinated and non-vaccinated animals.

Regarding dietary effect, no significant differences between treatment groups were observed for any subpopulation before infection (Figure S2, 0 dpi). After challenge infection, significant differences were found at single time points. Higher CTL percentages (P<0.05) were found in the Zn^high^+VAC group compared to the control Zn^low^+VAC group at 5 dpi. In non-vaccinated groups, higher CD4^+^CD8^+^ T cell percentages (P<0.05) were found in the *E. faecium* group compared to the Zn^low^ group at 2 dpi. Finally, significantly lower antibody-producing and/or memory B cell numbers were observed in the *E. faecium* group compared to the Zn^low^ group (P<0.05) at 1 dpi in non-vaccinated pigs.

Immune cell phenotypes of BAL cells were examined after necropsy on 1 dpi and 6 dpi. We observed an increase in percentages of gamma delta T-cell, activated B-cell and activated T-cell at 6 dpi after infection compared to 1 dpi, but no influence of vaccination and dietary treatment (Figure S3).

## Discussion

In this study, we investigated effects of feed supplementation with *E. faecium* or higher dietary ZnO levels on vaccination against and challenge with swine influenza A virus. Clinical follow-up, virological outcome, as well as humoral immune and cellular immune responses were recorded analyzed. To our knowledge, such information is the first to be collected and described in pigs, or any livestock, and, therefore, provides an important contribution with respect to the assessment of the usefulness, or lack thereof, of feed supplementation on an important viral disease.

Challenge infection with H3N2 SIV caused mild symptoms, which is in line with observations from other studies [25] and confirms the importance of good sanitary status, as provided during the experimentation here, in the prevention of secondary infections. The observation of significantly higher body weight gains in the *E. faecium* treatment groups after weaning and before infection was also made in other studies [12], [26], whereas a growth-promoting effect of the Zn^high^ diet as observed by others [26]–[28] could not be confirmed. Comparing body weights after challenge infection, it appeared that mean body weights of piglets in all Zn^high^ and *E. faecium* increased from 1 dpi to 6 dpi, while it decreased in the Zn^low^ groups (Figure S1B). These results might indicate a better and faster recovery from infection and anorexia of reduced duration in the probiotic and Zn^high^ groups.

The most prominent finding obtained in this study was the development of higher SIV-specific ELISA and HI antibody levels in the Zn^high^ and *particularly E. faecium* treated vaccine groups two days before as well as on the day of virus infection (Figures 6 and 7). The increased antibody response to vaccination in the group receiving the higher Zn level diet compared to the Zn^low^ diet group might indicate that a suboptimal Zn supply in the Zn^low^ group was restored, since it has been shown that a Zn deficiency impairs B-cell function [29]. It is also possible that a normal antibody response in the Zn^low^ group was improved by the additional Zn supply, although this has not been shown elsewhere yet. The data also demonstrate that dietary supplementation with *E. faecium* was able to boost antibody levels. Similar observations were also made in a previous study using another probiotic strain [30]. However, we and others can only speculate about possible mechanisms of how antibody titers to a vaccine applied parenterally might be enhanced by oral probiotics. We applied the influenza vaccine intramuscularly and we assume that immune responses were mainly generated in the tributary (axillary) lymph nodes. Some communication must, therefore, exist between probiotic bacteria in the gut and the cells initiating immune responses at a distant site to explain the observed effect. It was previously argued that (subcellular) fragments of probiotics may enter the bloodstream and as such have a very direct albeit weak adjuvant effect at a distant lymph node [11]. Another possible explanation could be that during feed intake some probiotic fragments might be inhaled and/or directly get in contact with epithelial cells in the nasopharynx and induce cytokines or other signaling molecules with an adjuvant effect. Interestingly the *E. faecium* group diet was based on the Zn^low^ diet, thus, not only could a possible lack of Zn be compensated by the probiotic supplement but also there could be a possible synergistic effect between *E. faecium* and optimal or elevated Zn for the induction of even higher antibody levels.

Lungs from non-vaccinated animals showed more extensive macroscopical lesions (Figures 2 and 3) than those from non-vaccinated animals. Microscopical evaluation revealed that vaccination reduced the severity of microscopic lesions (Figure 4). However, a dietary influence on these observations was not apparent.

Our data also shows that vaccination did not result in sterile immunity but reduced the number of animals shedding virus as well as the amount of virus shed from the nose and buccal sites (Figure 5). Fecal shedding was also tested but, in agreement with the literature [31], no virus could be detected. Despite higher antibody levels, a stronger reduction of virus shedding was not achieved by *E. faecium* or Zn^high^ supplementation in vaccinated animals. As reported by others [32], [33], an increase in antibody levels does not necessarily mean that these antibodies exhibit high specificity or affinity. This is especially true for antibodies induced by inactivated vaccines where, unlike following live vaccine administration or natural infection, virus is not delivered to secondary lymphatic organs and presented by dendritic cells to elicit optimal virus-neutralizing antibody responses.

Hematology revealed transiently reduced neutrophil numbers in both vaccinated and non-vaccinated animals receiving the Zn^high^ diet. Zn-induced neutropenia has been described in man [34]. Obviously, in this study, neutrophil numbers were still sufficient to avoid negative effects on the course of SIV infection. It needs to be emphasized that the Zn^high^ diet was reduced to a Zn^med^ diet (150 ppm) before infection to reduce the possibility of toxic effects. We, therefore, cannot rule out that, if continued, the high Zn doses might have had negative effects on health of the individuals.

According to the literature on cellular immune responses, CD4^+^ and CD8^+^ T cells as well as antibody-producing B cells make an important contribution to the control of influenza virus replication and virus clearance during infection [35], [36]. Th cells primarily stimulate antibody and cytokine production and proliferation of CTLs. The CTL response is mainly directed against the more conserved influenza virus proteins, M and NP. Consequently, a robust CTL response can also confer protection against heterologous influenza A virus challenge [35], [37]. Inactivated vaccines are poor inducers of cellular immune responses [38], and, accordingly, we observed no effect of vaccination on cellular immune responses. We found a slight decrease in Th cells and a concomitant equally slight increase of CTL and antibody-producing B cell percentages from 1 to 6 dpi in PBMCs. Regarding dietary effects, we found significant differences between the *E. faecium* and Zn^high^ groups and the control Zn^low^ group only at single time points but no prolonged effect. We also compared the percentages of immune cell phenotypes in cells of the BAL fluid after necropsy, since the proliferation responses in peripheral blood does not fully reflect those at the site of infection [39]. We found increased percentages of gamma delta T-cells, activated B-cells and activated T-cells at 6 dpi compared to those on 1 dpi in vaccinated and non-vaccinated animals, but no influence of dietary treatment. Thus it seems that *E. faecium* and Zn supplementation neither systemically nor locally changed the cellular immune response to SIV infection substantially.

In summary, the results presented here suggest that high doses of ZnO and particularly *E. faecium* can increase humoral immune responses following SIV vaccination and support recovery from clinical illness. However, the increased responses do not significantly affect virus shedding or the development of lung lesions after challenge infection. However, if used in combination with an appropriate vaccine, feed supplementation with ZnO and/or *E. faecium* might be suitable to improve antibody responses and to help reducing virus shedding.

## Supporting Information

Figure S1 **Animal weight analyses.** **(A)** Mean weekly weight gain before virus infection. Each bar represents the mean value ± standard deviation from 12 pigs (**: P<0.01. ***: P<0.001). **(B)** Mean body weights on the indicated day after virus. Weights were measured after exsanguination.
(TIF)

Figure S2 **Comparison of immune cell subsets of PBMCs.** Percentages of Th cells **(A, B)**, CTLs **(C, D)**, Th/memory cells **(E, F)**, antibody-forming/memory B cells. **(G, H**) and NK cells **(I, J)** in PBMCs from day 0 to 6 dpi. Each bar represents the mean value ± standard deviation from 6 pigs (*: P<0.05).
(TIF)

Figure S3 **Comparison of immune BAL cell subsets.** Percentages of Th cells (CD4^+^CD8^−^); CTLs (CD4^−^CD8^+^); Th/memory cells (CD4^+^CD8^+^); gamma delta T cells (CD2^+^CD8^+^); antibody-producing and/or memory B cells (CD2^+^CD21^−^); activated Th cells (CD8^−^CD25^high^), and NK cells (CD3^−^CD8^high^) at 1 dpi and 6 dpi in vaccinated (upper panel) and non-vaccinated (lower panel) animals.
(TIF)

Table S1 **Primary and secondary antibodies used for flow cytometry staining.**
(DOCX)
