# Supplementary figures and images for: Dietary Enterococcus faecium NCIMB 10415 and Zinc Oxide Stimulate Immune Reactions to Trivalent Influenza Vaccination in Pigs but Do Not Affect Virological Response upon Challenge Infection
Source: PLoS One. 2014 Jan 28;9(1):e87007. doi: 10.1371/journal.pone.0087007 (PMC3904981; doi:10.1371/journal.pone.0087007)

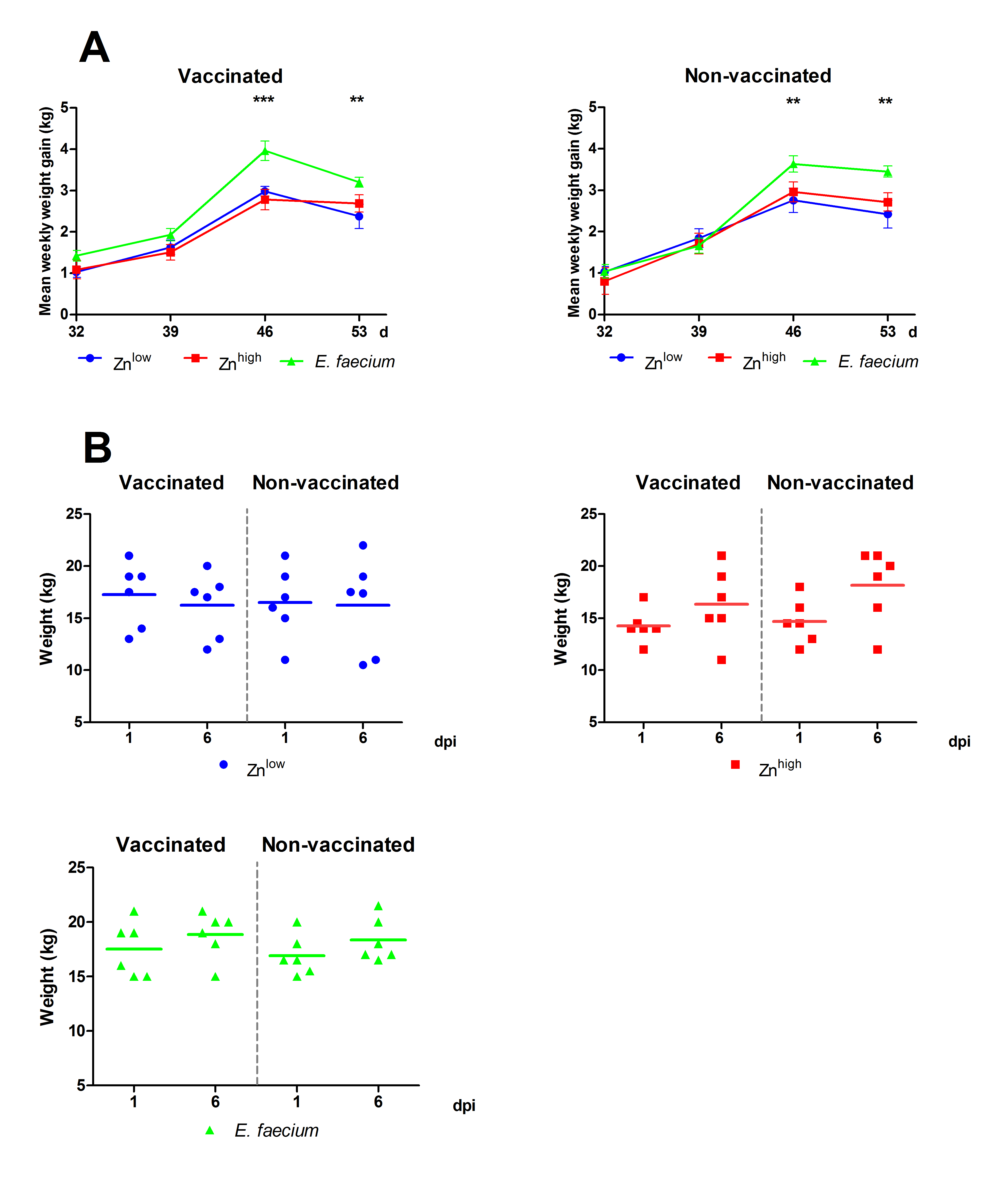

Supplement: Figure S1 — Animal weight analyses. (A) Mean weekly weight gain before virus infection. Each bar represents the mean value ± standard deviation from 12 pigs (**: P<0.01. ***: P<0.001). (B) Mean body weights on the indicated day after virus. Weights were measured after exsanguination. (TIF) [file pone.0087007.s001.tif]

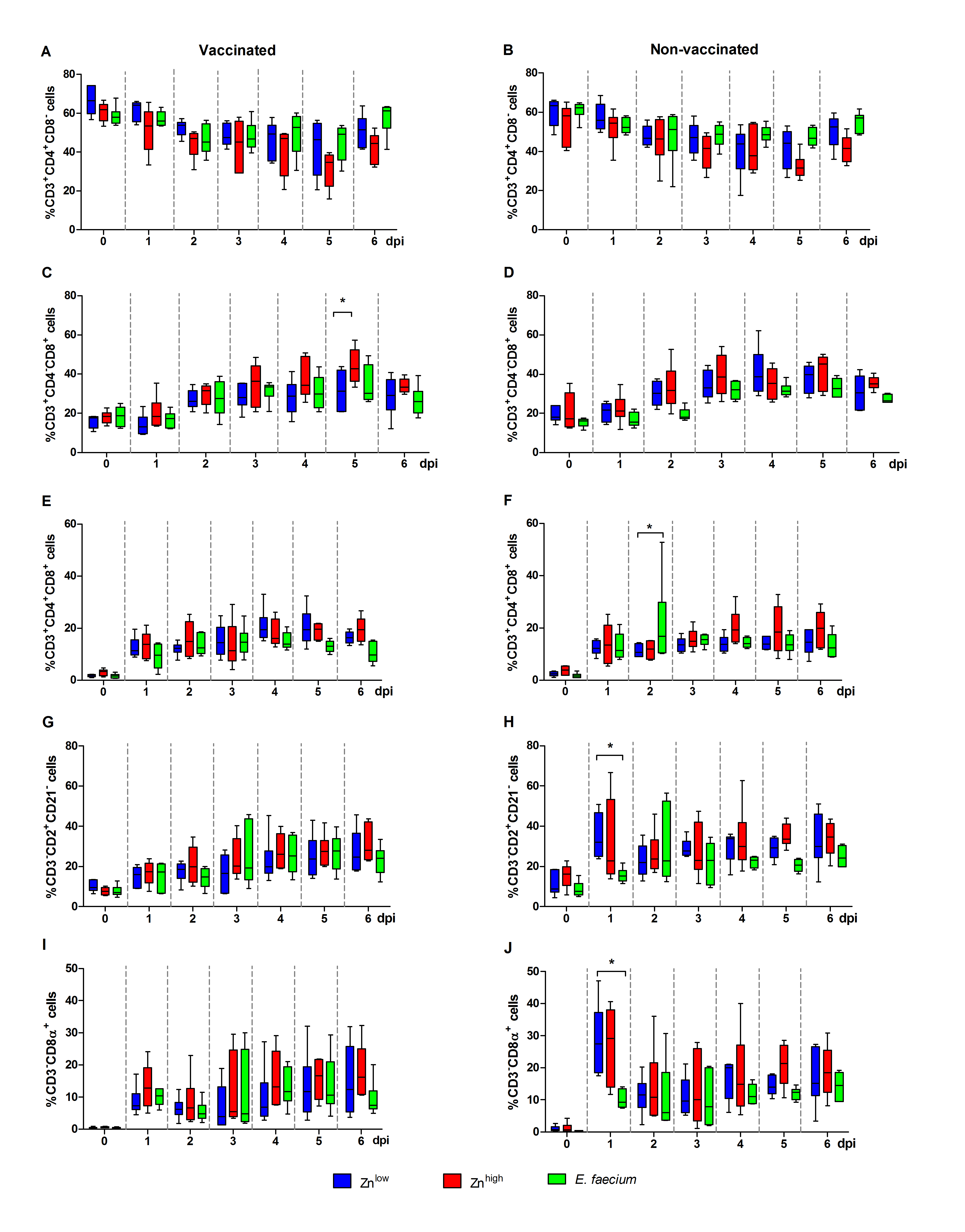

Supplement: Figure S2 — Comparison of immune cell subsets of PBMCs. Percentages of Th cells (A, B), CTLs (C, D), Th/memory cells (E, F), antibody-forming/memory B cells. (G, H) and NK cells (I, J) in PBMCs from day 0 to 6 dpi. Each bar represents the mean value ± standard deviation from 6 pigs (*: P<0.05). (TIF) [file pone.0087007.s002.tif]

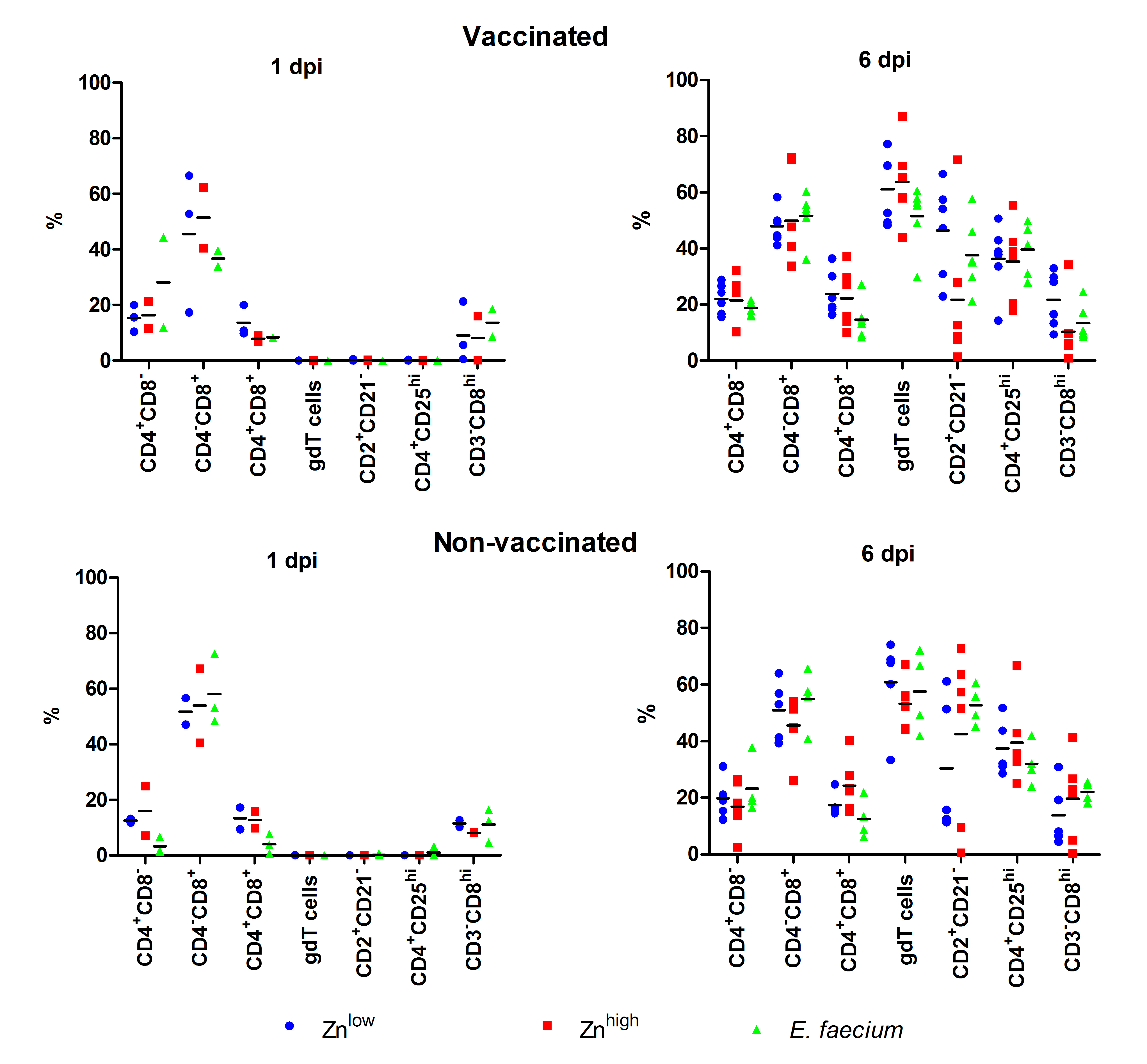

Supplement: Figure S3 — Comparison of immune BAL cell subsets. Percentages of Th cells (CD4+CD8−); CTLs (CD4−CD8+); Th/memory cells (CD4+CD8+); gamma delta T cells (CD2+CD8+); antibody-producing and/or memory B cells (CD2+CD21−); activated Th cells (CD8−CD25high), and NK cells (CD3−CD8high) at 1 dpi and 6 dpi in vaccinated (upper panel) and non-vaccinated (lower panel) animals. (TIF) [file pone.0087007.s003.tif]
