# Supplementary material for: Dietary Enterococcus faecium NCIMB 10415 and Zinc Oxide Stimulate Immune Reactions to Trivalent Influenza Vaccination in Pigs but Do Not Affect Virological Response upon Challenge Infection
Source: PLoS One. 2014 Jan 28;9(1):e87007. doi: 10.1371/journal.pone.0087007 (PMC3904981; doi:10.1371/journal.pone.0087007)
Supplement: Table S1 — Primary and secondary antibodies used for flow cytometry staining. (DOCX) [file pone.0087007.s004.docx]

**Table S1. Primary and secondary antibodies used for flow cytometry staining**

| **Mix** | **Antibody** | **Isotype** | **Labeling** | **Clone** | **Source** |
| --- | --- | --- | --- | --- | --- |
| 1 | Mouse anti-pig CD2 | IgG2a | None | MSA 4 | Hybridoma supernatant |
|  | Mouse anti-pig CD25 | IgG1 | None | K231.3B2 | AbD Serotec |
|  | Mouse anti-pig TCRγδ | IgG2b | None | PPT 16 | Hybridoma supernatant |
| 2 | Goat anti-mouse IgG1 | IgG1 | DyLight^®^ 405 | polyclonal | Dianova |
|  | Goat anti-mouse IgG2a | IgG2a | APC-Cy^™^7 |  | Southern Biotech |
|  | Goat anti-mouse IgG2b | IgG2b | PE | polyclonal | Dianova |
| 3 | Mouse anti-pig CD3 | IgG2a | Alexa Flour^®^648 | BB23-8E6-8C8 | BD Pharmingen |
|  | Mouse anti-pig CD4 | IgG2b | PerCP-Cy^™^5.5 | 74-12-4 | BD Pharmingen |
|  | Mouse anti-pig CD8α | IgG2a | FITC | 76-2-11 | Southern Biotech |
|  | Mouse anti-pig CD21 | IgG1 | Biotin | BB6-11C9.6 | Southern Biotech |
| 4 | Streptavidin |  | PE-Cy^™^7 |  | Southern Biotech |
